# Overland and oversea migration of white storks through the water barriers of the straits of Gibraltar

**DOI:** 10.1038/s41598-020-77273-x

**Published:** 2020-12-01

**Authors:** Julio Blas, Reyes Salas, Andrea Flack, Fernando Torres-Medina, Fabrizio Sergio, Martin Wikelski, Wolfgang Fiedler

**Affiliations:** 1 Department of Conservation Biology. Estación Biológica de Doñana, Consejo Superior de Investigaciones Científicas CSIC, 41092 Seville, Spain; 2 Department of Migration, Max Planck Institute of Animal Behavior, 78315 Radolfzell, Germany; 3 Centre for the Advanced Study of Collective Behaviour, University of Konstanz, 78457 Konstanz, Germany

**Keywords:** Ecology, Animal migration, Behavioural ecology

## Abstract

Soaring landbirds typically exploit atmospheric uplift as they fly overland, displaying a highly effective energy-saving locomotion. However, large water bodies lack thermal updrafts, potentially becoming ecological barriers that hamper migration. Here we assessed the effects of a sea surface on the migratory performance of GPS-tagged white storks (*Ciconia ciconia*) before, during and after they crossed the straits of Gibraltar. Oversea movements involved only flapping and gliding and were faster, traversed in straighter, descending trajectories and resulted in higher movement-related energy expenditure levels than overland, supporting the water barrier hypothesis. Overland movements at both sides of the sea straits resulted in tortuous routes and ascending trajectories with pre-crossing flights showing higher elevations and more tortuous routes than post-crossing, thus supporting the barrier negotiation hypothesis. Individual positions at both ends of the sea narrow were predicted by zonal winds and storks´ location at entry in the European hinterland, and birds did not show compensational movements overland in anticipation to subsequent wind displacements oversea. The length of the water narrow at departure shore, the elevation therein and the winds on route affected major components of sea crossing performance (such as distances and times overwater, minimum elevations, climb angles, speeds and energy expenditure), supporting the departure position and oversea winds hypotheses. In summary, our study provides a prime example at high temporal resolution of how birds adjust their behavior and physiology as they interact with the changing conditions of the travelling medium, reallocating resources and modifying their movement to overcome an ecological barrier.

## Introduction

The evolution of terrestrial soaring has allowed birds to maximize their movement capabilities overland while minimizing energy costs^1,2^, with large, broad-winged landbirds such as storks and raptors mastering this type of flight^3,4^. By exploiting the rising air currents around uplift hotspots generated by sun-heated surfaces and orography, soaring landbirds elevate until reaching a preferred height, and then glide between sequential hotspots thus reducing energy-demanding wing flapping^5,6^. Since the atmosphere yields most of the energy required for this type of flight, the movement ecology of soaring landbirds is largely constrained by geography, sun and weather conditions ultimately determining uplift (e.g., thermals are generally lacking overwater, overnight and under inclement weather). As a consequence, large water bodies such as rivers, lakes, seas and oceans can hamper the flight of soaring landbirds, potentially becoming natural barriers to movement that divert and shape migration routes^7–9^. For example, the migratory movements of white storks (*Ciconia ciconia*) between Europe and Africa are largely constrained by the Mediterranean sea, which diverts migrations into Western and Eastern routes, respectively funneled through the sea straits of Gibraltar and Bosporus^10,11^ where birds can be hesitant to initiate sea crossings, frequently wandering near the coast and often showing failed crossing attempts^3,12,13^. Understanding whether and how animals adjust their behavior as they overcome ecological barriers is a topic of major interest to ecologists and evolutionary biologists, because (i) travelling animals are interacting with an environment that can be hostile and dangerous, (ii) their behavior and physiology may be pushed to their limits, and therefore (iii) natural selection has a strong potential to operate readily penalizing suboptimal choices^14,15^.

Historically, naturalists and ecologists have shown substantial interest in the study of avian movements through migratory bottlenecks like the straits of Gibraltar (e.g.^12,16,17^), which annually funnels many millions of birds, including several hundred thousands of storks and raptors^8,18,19^. However, traditional research has largely relied on visual observations of complete flocks from the hinterlands, precluding continuous individual monitoring over large scales and thus leaving many ecological questions unresolved. The use of radar tracking technology and more recently, the advent of lightweight, global tracking, onboard sensors is allowing to monitor animal behavior remotely, individually and over large spatial scales (e.g.,^3,20,21^). As a consequence, it is now possible to test with unprecedented resolution a number of hypotheses such as those summarized in Table 1, regarding the movement of soaring landbirds before, during and after crossing large water bodies. Avian movements through sea straits can be generally framed within a "water barrier hypothesis", which posits that large bodies of water constrain the flight performance and increase the physiological demands of soaring landbirds^7,9,13,22–24^. From a proximate perspective, the lack of solid ground implies a lack of both convective updrafts and safe, predictable landing surfaces which may increase flapping effort, the risk of fatigue and the possibility of being blown out to sea due to crosswinds and deteriorating weather conditions, potentially leading to drowning (e.g., Zu-Aretz & Leshem report more than 1,300 dead raptors on a beach^14^; see also^25^). This framework of the water barrier hypothesis predicts that overwater movements will involve reduced or suppressed soaring and thus faster speeds and straighter routes, resulting in descent trajectories and higher movement-related energy demands compared to overland flights^13,22,23^.

**Table 1:** Summary of the hypotheses and predictions tested within the frame of this study analyzing storks’ movements before, during and after they migrated though a water narrow.

Hypotheses	Predictions
**Water barrier (framework hypothesis):** water bodies constrain the flight performance of soaring landbirds and increase their movement-related physiological demands	Overwater movements will involve (i) reduced or suppressed soaring, (ii) faster speeds, (iii) straighter routes, (iv) descent trajectories and (v) higher ODBA levels compared to overland movements
**Barrier negotiation:** overland movements pre-cross are exploratory and preparatory for the sea crossing	Pre-cross movements overland will be (i) more tortuous, (ii) slower and (iii) reaching higher altitudes compared to post-cross movements overland
**Anticipatory compensation**: birds anticipate already overland the subsequent wind displacement they may experience oversea and compensate accordingly their position at departure shore	Pre-cross movements seawards will show active compensation responses, opposite and proportional to the prevailing zonal winds (e.g., easterlies will promote eastern sea departures aimed at compensating the expected westward drag oversea)
**Departure position**: the specific position at departure shore exerts major consequences on sea crossing performance	Choosing departure locations that shorten the length of the water narrow and increasing flight altitude at departure will facilitate sea crossing performance (e.g., reducing travel distance, time and ODBA, and increasing climb and minimum altitude above water)
**Oversea winds**: the wind components experienced overwater exert major consequences on sea crossing performance	Tail winds will facilitate sea crossing performance whereas cross winds and head winds will jeopardize sea crossings (e.g., tail winds will decrease air speed and air distance, and cross winds will exert the opposite effects)

Within this general framework, several offshoot hypotheses can be posited. For example, obligate soaring landbirds typically wander along the coast before attempting to cross the sea and often abort initiated crossings^12,15,23,26^, suggesting that overland movements through the hinterland are exploratory and preparatory for barrier crossing ("barrier negotiation hypothesis", Table 1). According to the barrier negotiation hypothesis, we may expect that pre-cross itineraries will be more tortuous, slower and aimed at reaching higher altitudes compared to post-cross movements overland. Also, migratory bottlenecks typically occur at the narrows of large water bodies oriented perpendicular to preferred routes of travel^8,27^ where crosswinds increase travel distance, flapping effort, the probability of quitting sea crossing attempts and the possibility of being blown out to open waters^13,15,23,26^, opening the possibility that birds anticipate already overland the subsequent wind displacement they may experience oversea and perform adaptive adjustments of their position at the departure shore ("anticipatory compensation hypothesis", Table 1). The migration of soaring landbirds across the straits of Gibraltar occurs in a north–south axis^12^ and therefore, anticipatory compensation might be expected during pre-cross movements seawards^19^, opposite and proportional to the prevailing zonal winds (e.g., easterlies will promote eastern sea departures aimed at compensating the expected westward drag oversea). On a more practical level, the analysis of pre-cross movements may allow predicting individual location at both sides of the sea barrier, aiding visual observers to monitor migration more accurately^19^. The adaptive significance of barrier negotiation and anticipatory compensation rely on the potential consequences that the specific position chosen by the birds at the departure shore may exert on subsequent sea crossing performance. In this regard, the "departure position hypothesis" (Table 1) predicts that choosing departure locations that both shorten the length of the water narrow and elevate departure altitude will facilitate oversea crossing by means of reducing traversed distance, travel time and movement-related energy investment, and increasing the climb and minimum altitude above water^12,19,23^). Finally, the wind conditions experienced through the water barrier likely exert major consequences on sea crossing flight performance ("oversea winds hypothesis", Table 1). In particular, we may expect that tail winds will facilitate sea crossing (e.g., decreasing travel time, air speed, air distance and movement-related energy investment) whereas cross winds and head winds will exert the opposite effects^15,23,28^. To test these hypotheses, we monitored the movements of European white storks (*Ciconia ciconia,* thereafter "storks") tagged with miniature, solar, GSM (Global System for Mobile Communications), high-resolution GPS (Global Positioning System) and accelerometers (ACC) as they migrated from Europe to Africa through the straits of Gibraltar.

## Results

### Flight performance oversea compared to overland, and pre-cross compared to post-cross

**Figure 1:**
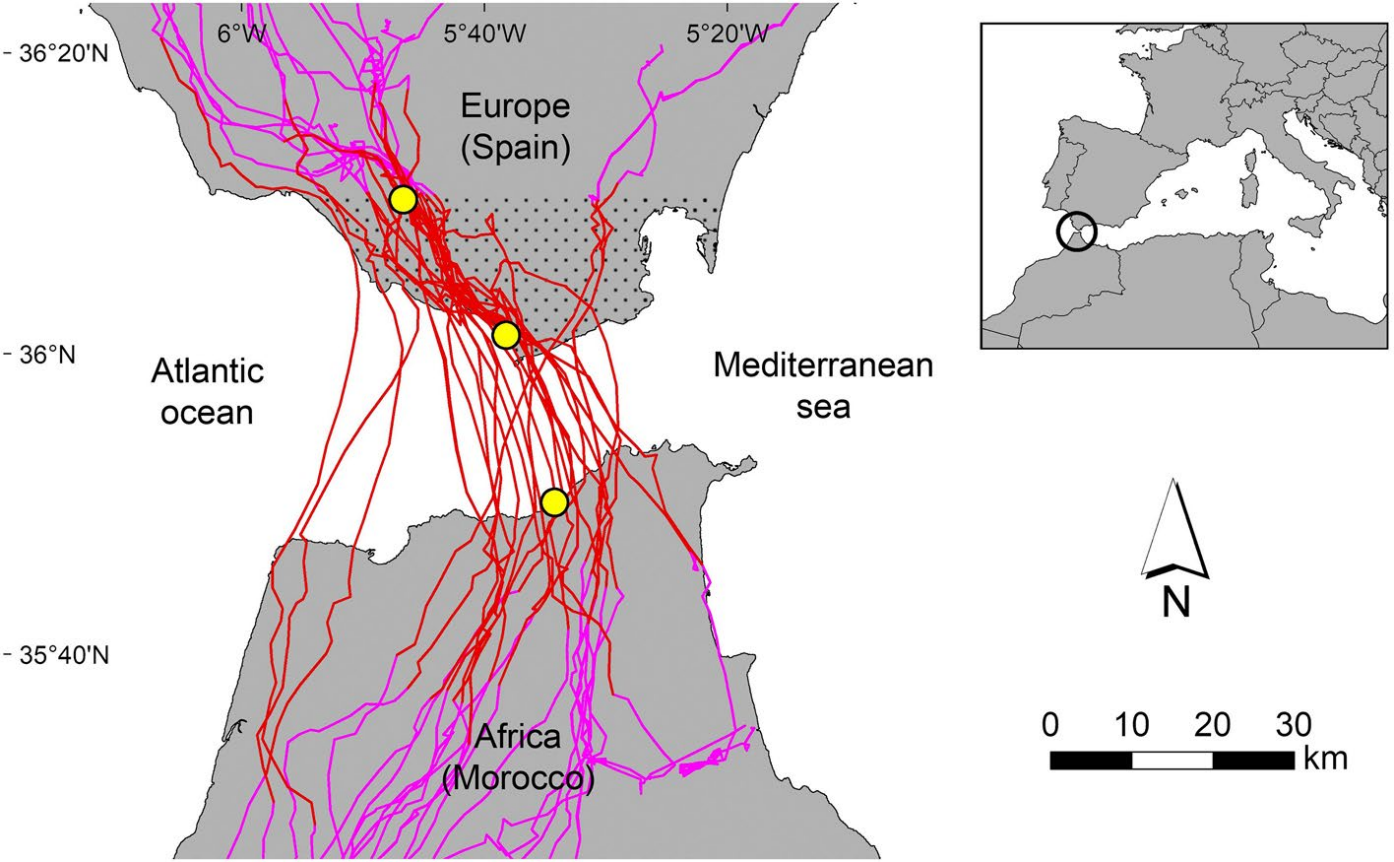
Study area and stork movement tracks. Overland and overwater movements of individual white storks during post-breeding (southbound) migration across the straits of Gibraltar. Red lines depict the itineraries considered for the comparison of overland vs oversea flight performance, and magenta lines show other portions of the same-day tracks. The dotted area depicts the European hinterland, and the yellow circles show the average population position across three sequential migratory milestones, namely: entry to the European hinterland pre-cross, departure from the European shore, and arrival at the African shore post-cross. Map generated with ArcGIS 10.5 software (ESRI, Redland, USA), country limits background generated from GDAM data (freely available for academic use in https://gadm.org/data.html), and composition assembled with Adobe Photoshop CS3 software.

**Figure 2:**
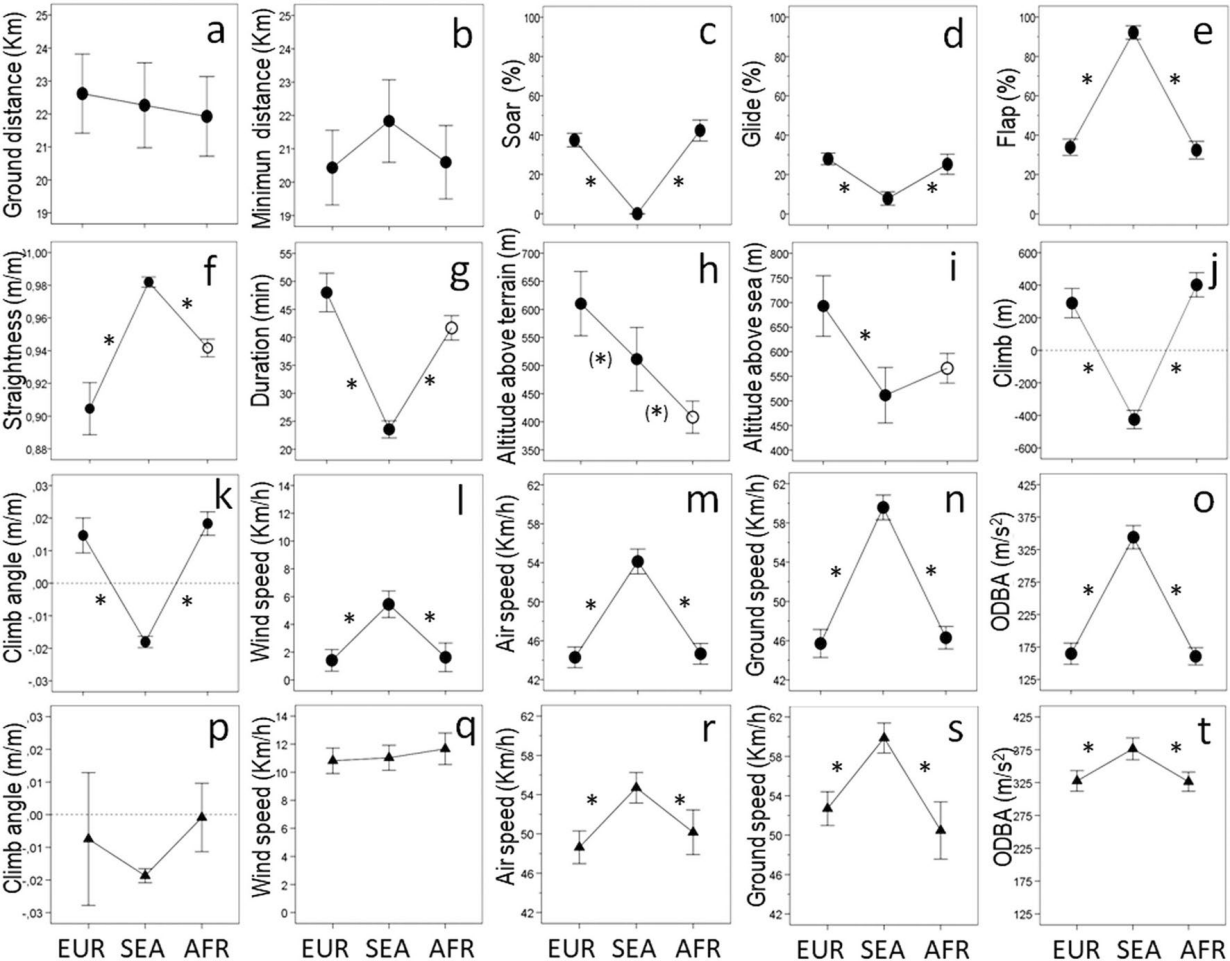
Sequential changes in trip and flight parameters across the overland and overwater itineraries (X-axis) comprising storks´ southbound migration through the straits of Gibraltar, namely: overland itineraries traversed pre-cross in the European shore (EUR), overwater itineraries across the sea straits of Gibraltar (SEA) and overland itineraries traversed post-cross, upon arrival at the African shore (AFR). Asterisks * indicate statistically significant differences (P < 0.05) and asterisks within brackets (*) indicate marginally significant differences (0.05 < P < 0.07) between sequential itineraries. Open circles depict statistically significant differences between AFR and EUR. Circles indicate parameters calculated using all the GPS fixes within a given itinerary, and triangles indicate parameters calculated using only GPS fixes associated to flapping flight. Error bars represent standard errors.

Descriptive statistics of the main trip and flight parameters characterizing the oversea and overland itineraries through the straits of Gibraltar (Fig. 1) are provided in Table S1. Birds showed a drastic behavioral shift from a mixed flight mode typically displayed overland (involving soaring, gliding and flapping in roughly similar proportions), to flights dominated by wing flapping and gliding with a virtual suppression of soaring across the sea straits (Figs. 2c–e, S1). Although the sampled distances were similar among itineraries (Fig. 2a–b), oversea routes were straighter and were traversed in shorter times than terrestrial itineraries (Fig. 2f–g). Pre-cross itineraries overland were less straight and were traversed in longer times than post-cross itineraries overland (Fig. 2f–g, Table S2). The average flight altitude was highest over European grounds pre-cross, and declined afterwards (Fig. 2h–i, Table S2). Overland flights resulted in a net gain of altitude contrary to oversea flights, which resulted in negative climbs (Fig. 2j–k, Table S2). Storks travelled at higher speeds and displayed higher mean ODBA (overall dynamic body acceleration) levels across the sea straits compared to overland (Fig. 2m–o, Table S2). When we only considered flapping flight, the climb angles and wind speeds were similar overland and oversea, but the air speed, ground speed and mean ODBA levels were higher oversea (Fig. 2p–t, Table S3).

### Flight performance through the oversea itinerary

All flight parameters remained constant throughout the itinerary overwater except flight altitude (Fig. S2, Table S4). Birds started their oversea itineraries at 727 ± 73 m.a.s.l. (mean and s.e.m.; range 72–1504 m), experienced a gradual loss of elevation as they flew across the sea straits (Figs. S1 and S2) and displayed the lowest elevations as they reached the African shore at 302 ± 47 m.a.s.l. (mean and s.e.m.; range 22–864 m).

### Causes and consequences of route choices

**Figure 3:**
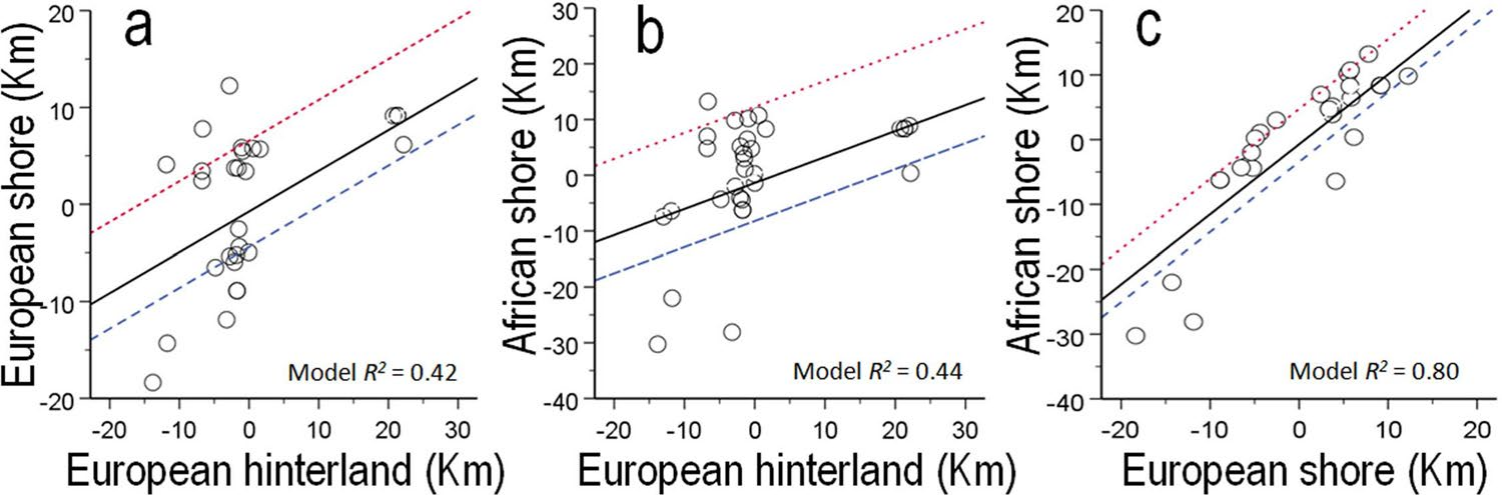
Location of storks at both ends of the straits of Gibraltar (i.e. European shore at departure, and African shore upon arrival) in relation to their previous locations and concurrent zonal winds during southbound migration. Location represents east or west deviations (positive and negative values respectively) from the mean population longitude at the three milestones characterizing oversea migration routes: the European hinterland, the European (departure) shore, and the African (arrival) shore. Circles represent observed data and lines represent model predictions under three illustrative zonal wind scenarios: westerlies (dotted red lines; 6 m/s), lack of zonal winds (filled black line; 0 m/s) and easterlies (dashed blue lines; 3 m/s). The location of birds when they entered the European hinterland and the concurrent zonal winds therein predicted storks´ subsequent location in the European shore (**A**) and in the African shore (**B**). The latter was best predicted storks´ location in the European shore and the concurrent winds therein (**C**).

Storks entered the European hinterland across a 36 km-wide longitudinal front (Fig. 1), and traversed seawards along 24.3 ± 29.8 km (mean ± s.e.m.; range 7.7–94.0) during 52 ± 7 min (range 20–225) before departing to Africa. All but two birds succeeded to cross the water channel at their first oversea flight. The two aborted sea crossing attempts were followed by additional overland movements during 10–190 min until reaching a final shore departure location. Sea departures occurred through a continuous front spanning 40 km of the European shoreline, with the average and the median departure longitudes being located near the narrowest water-crossing point (i.e. 4 km West and 1 km East of the Tarifa isthmus respectively). Storks traversed overwater 22.3 ± 1.3 kms (range 15.1–38.2) during 23.5 ± 1.5 min (range 14–44) until reaching African grounds through a continuous front spanning 51 km of shoreline (Fig. 1). The location of storks when they entered the hinterland and the concurrent zonal wind velocity therein were able to predict the subsequent locations of birds at departure in the European shore, and at arrival in the African shore (Fig. 3a,b; Table S5). The location of arrival at the African shoreline was also predicted by prior departure location on the European shore and concurrent zonal wind components (Fig. 3c; Table S5), and neither meridional wind components nor the interaction between winds and location exerted significant effects (i.e. all P > 0.05).

### Factors affecting flight performance oversea

**Figure 4:**
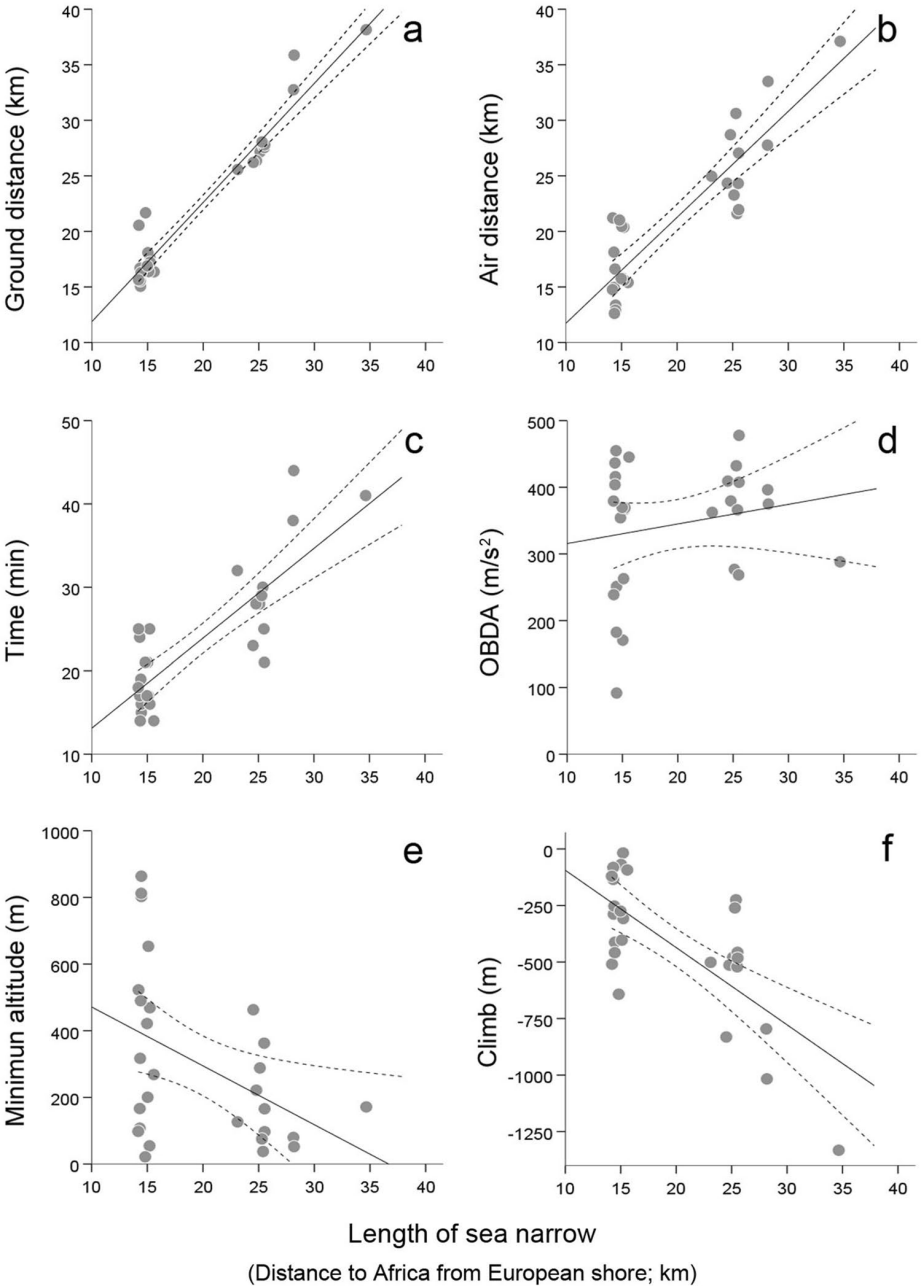
Effects of the length of the sea narrow at departure shore (i.e. distance between Europe and Africa at the starting point of the sea crossing) on several parameters defining flight performance oversea. Solid and dashed lines respectively represent predicted effects based on partial regression models and 95% mean confidence intervals. Dots represent recorded observations.

**Figure 5:**
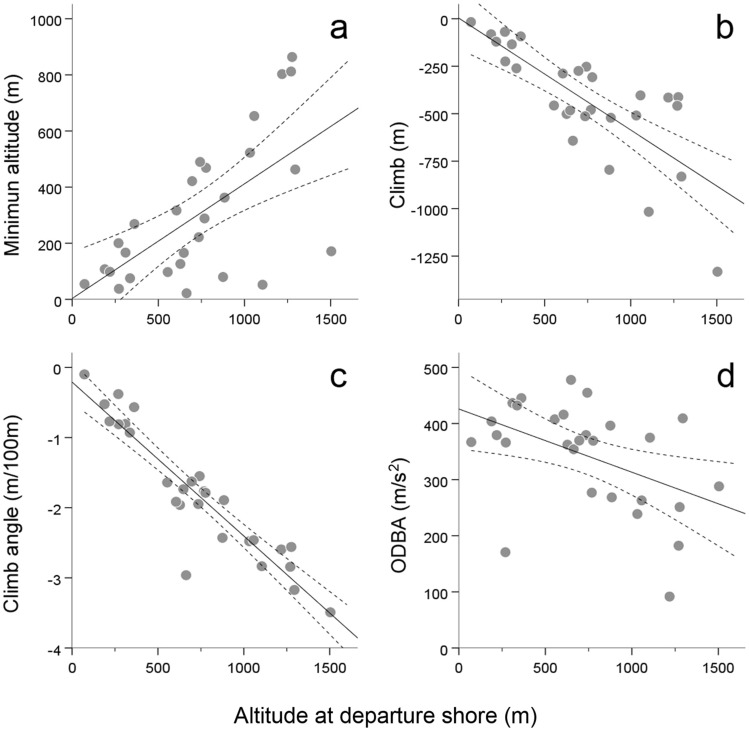
Effects of the elevation at departure shore in Europe (at the starting point of the sea crossing towards Africa) on several parameters defining flight performance oversea. Solid and dashed lines respectively represent predicted effects based on partial regression models and 95% mean confidence intervals. Dots represent recorded observations.

The length of the water narrow at departure shore positively affected ground distance, air distance, time and mean ODBA overwater, and negatively affected climb and the minimum elevation reached across the oversea itinerary (Fig. 4; Tables 2 and S6). The elevation at departure shore decreased ODBA, climb and climb angle, and increased the minimum elevation experienced oversea (Fig. 5; Tables 2 and S6). Wind support reduced air distance and air speed, and cross winds increased air speed oversea (Fig. 5; Tables 2 and S6).

**Table 2:** Summary of results from the Generalized Linear Models GLM (A-I) analyzing sea crossing performance as a function of departure position at the start of the oversea itinerary and wind conditions experienced on route.

	Flight components oversea (response variables)	Tested effects			
		At departure		On route	
		Length of sea narrow	Departure altitude	Wind support	Cross wind^2^
A	Ground distance oversea	+	·	·	·
B	Air distance oversea	+	·	−	·
C	Time oversea	+	·	·	·
D	Ground speed oversea	·	·	·	·
E	Air speed oversea	·	·	−	+
F	Mean ODBA oversea	(+)*	−	·	·
G	Minimum altitude oversea	−	+	·	·
H	Climb oversea	−	−	·	·
I	Climb angle	·	−	·	·

Statistically significant effects are denoted with + and − signs (indicating positive and negative effects respectively). Details on the GLMs structure and results are provided in Table S6.

*Marginally significant effect (P = 0.059).

## Discussion

Our results showed the following patterns regarding storks´ movements before, during and after they crossed the water narrow of the straits of Gibraltar, that will be discussed in line with the hypotheses proposed in Table 1: (1) overland movements involving soaring, flapping and gliding at both sides of the sea straits resulted in tortuous routes and ascending trajectories; (2) pre-crossing flights were performed at higher elevations and along more tortuous routes compared to post-crossing; (3) oversea movements involved only flapping and gliding and were faster, traversed in straighter, descending trajectories and resulted in higher ODBA levels than overland; (4) when we only considered flapping flight, speed and ODBA remained higher oversea than overland; (5) individual positions at both ends of the sea narrow were predicted by zonal winds and storks´ location at entry in the European hinterland; and (6) the length of the water narrow at departure shore, the elevation therein and the winds on route affected major components of sea crossing performance (such as distances and times overwater, minimum elevations, climb angles, speed and ODBA).

### Water barrier hypothesis

Flight parameters showed a marked change when storks flew overwater compared to previous and subsequent oversea movements (Fig. 2). Supporting the water barrier hypothesis, oversea flights involved faster speeds, straighter routes through descending trajectories and higher ODBA levels than overland. Such changes were primarily a consequence of the constraints imposed by a travelling medium lacking thermal and orographic uplift, which forced storks to abandon soaring-gliding flight and rely on active wing flapping assisted by gliding as they loss elevation across the sea barrier (i.e. on average, storks crossed the straits of Gibraltar by climbing to above mean altitude and gliding at above mean distance assisted by flapping; Figs. 2 and S1). Air speeds displayed a mean 22% increase (with mean and peak values of 54 km/h and c.a. 80 km/h, respectively) from overland pre-cross to oversea itineraries. When we restricted speed comparisons to flapping flight, air speed still showed a significant 13.6% increase with mean and peak values of 55 km/h and 66 km/h respectively overwater, suggesting that the increased speed was not solely a consequence of the lack of soaring and that storks increased flapping effort (see^29,30^ for explicit comparisons between flight speeds in soaring birds and theoretical predication of time vs. energy optimal speeds). Although some birds gained elevation at the early stages of the sea crossing (likely exploiting uplift currents originated in the hinterland, and displaced by north winds into the sea straits), storks displayed an overall descending trajectory across the sea channel (Figs. 2j–k and S1), loosing on average more than 400 m and up to 1300 m overwater and displaying elevations as low as 22 m.a.s.l. upon reaching African shores. The negative climb and reduced elevations oversea reflected the movement constraints of a species primarily evolved to soar over terrestrial environments, but also revealed an adaptive strategy where the storks´ energy potential was likely traded for kinetic energy to facilitate progression across the water barrier. Since the flapping climb angle was similar overland and overwater, the reported 13.6% increase in air speed could not be explained by a higher rate of conversion of energy potential into kinetic energy, thus suggesting a higher frequency of wing flapping (as recorded in Griffon vultures *Gyps fulvus*^15^) that would be consistent with the recorded 15% rise in ODBA while flapping oversea. In fact, when we compared complete itineraries, oversea tracks displayed an overall 108% rise in ODBA compared to overland. Given the tight links previously established between ODBA levels and movement-related energy expenditure, including avian flight^31–36^, our results empirically support the idea that migrating across sea straits imposes physiological burdens to white storks, reinforcing the view of water bodies as movement barriers and supporting the water barrier hypothesis^7,13,23,24,37,38^. Considering that overwater movements lasted half the time recorded in overland flights of similar lengths, the ODBA per distance was actually similar across itineraries, which nonetheless differed in ODBA per time unit thus revealing the higher power displayed overwater. The recorded flight parameters overwater suggested movement constraints that may allow us to infer and explain storks´ migratory patterns at geographical scales beyond our study settings. For example, storks from Central-European populations seem to avoid migration across Italy via the 140 km-wide strait of Sicily, thus detouring through Spain or Turkey at significant travel costs^11^. Considering the descent angle and air speeds we recorded overwater, migrating through the strait of Sicily may require storks to climb above 3000 m before departure from Europe, and then accomplish a single, non-stop, oversea flight for almost 3 h until reaching the closest shore in Africa. Unless wind conditions were extremely favorable, such parameters would probably impose a considerable physiological burden to white storks, increasing the risks of fatigue and of drifting off course due to cross winds, and explaining selection for long migratory detours^37^ like the Western route we monitored across the straits of Gibraltar.

### Barrier negotiation hypothesis

Overland itineraries involving soaring, gliding and flapping (Figs. 2 and S1) were consistent with a movement primarily oriented to locate and exploit atmospheric uplifts along the route^3,12,18^, explaining the higher tortuosity, longer travel times, slower speeds and moderate ODBA levels compared to overseas tracks where soaring was lacking. When we compared pre- and post-crossing itineraries of similar length overland, the route seawards displayed higher tortuosity, longer travel times and higher elevations (Fig. 2f–h). This pattern supported the barrier negotiation hypothesis that pre-cross movements required more exploration in search for adequate conditions at shore departure and a higher gain of energy potential in preparation for the sea crossing upon shore-departure^19,23,39^. Overland flights thus likely reflected a strategic movement for barrier negotiation pre-cross compared to and a less convoluted route at lower elevations post-cross.

### Anticipatory compensation hypothesis

**Figure 6:**
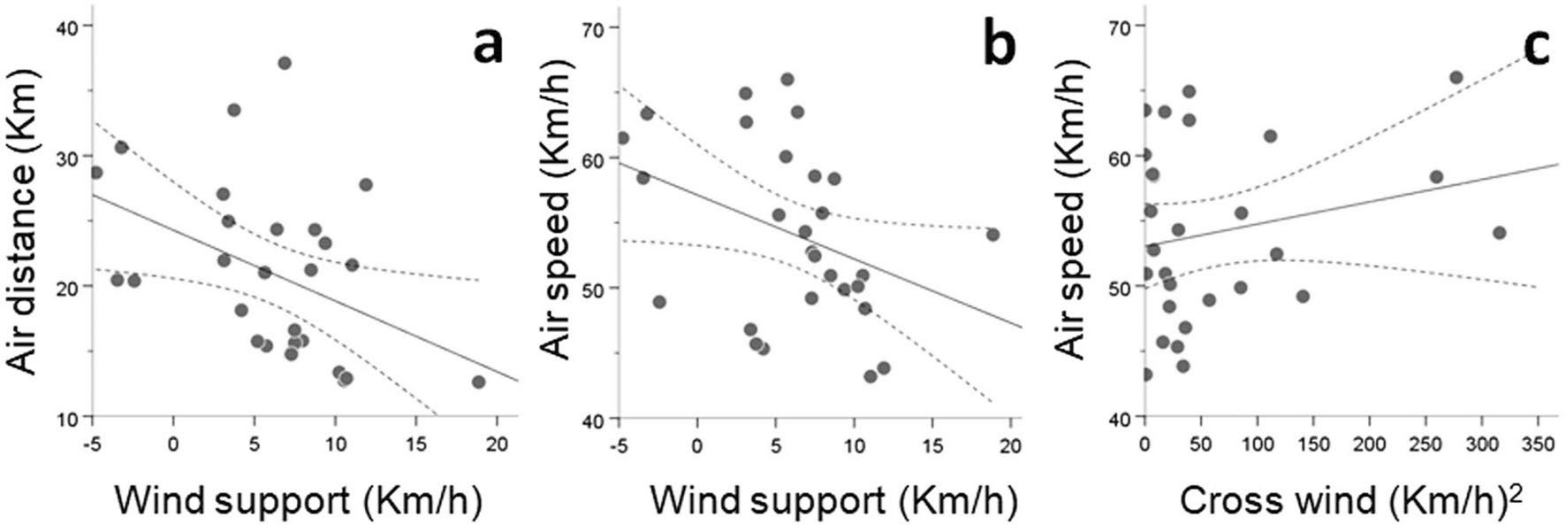
Effects of the wind components experienced during the sea crossing on several parameters defining flight performance overwater. Solid and dashed lines respectively represent predicted effects based on partial regression models and 95% mean confidence intervals. Dots represent recorded observations.

Storks´ departure and arrival locations at both sides of the sea barrier could be predicted by their position and concurrent winds when they entered the European hinterland (Fig. 3). Indeed, the latter two variables explained 42% of the recorded positions at departure shore, and 44% at arrival shore. Furthermore, departure locations in the European shore and concurrent zonal winds predicted 80% of the variability of locations at arrival to the African shore. Migratory routes across the water barrier thus likely resulted from the combined effects of predetermined individual route decisions before entering the hinterland, and subsequent adjustments to the prevailing zonal winds that passively displaced the birds before and during their ocean crossings. The latter result was contrary to the anticipatory compensation hypothesis and opposed to previous reports based on visual observations of storks at the study site^19^. Indeed, our GPS-tagged storks experienced a passive displacement rather than an active compensation movement in response to the prevailing zonal winds, showing a pattern that was consistent with the spatial changes in departures locally recorded in other soaring landbirds such as black kites *Milvus migrans*^23^. Within the conditions recorded in our study, itinerary straightness oversea was close to the maximum possible value (i.e. 98.2%) from the realized departure location of the birds, explaining the strong association (i.e. r^2^ = 0.80) between individual positions at both ends of the sea narrow (Figs. 3c, 1). The largely parallel and rarely intercrossing oversea tracks we recorded are likely a consequence of the relatively mild winds (i.e. below 25 km/h) experienced within the course of our study, which are representative of the environmental conditions characterizing storks´ sea crossings (i.e. 80% of storks’ sea crossings occur with wind speeds ranging 5–25 km/h, and virtually disappear with wind speeds over 50 km/h^19^). Stronger winds are frequent in the study area^40^ and particularly strong easterlies above 30–60 km/h have been shown to restrict the passage of soaring birds to Africa^19,23,28^. However, we anticipate that winds speeds faster than those recorded during our study would produce less parallel and more intercrossing trajectories, partly explained by storks´ aims at increasing wind support and reducing air distance oversea. Such prediction would be consistent with our results showing that zonal winds passively displaced storks before and during their ocean crossings (Fig. 3) and that wind support significantly reduced air distance oversea (Fig. 6).

### Departure position and oversea winds hypotheses

Our results also showed that major components of the flights oversea were determined by the starting position at departure shore and wind conditions (Table 2). Departure locations determined the length of the sea narrow, and the latter variable alone explained travel distance and time overwater. By means of choosing a short sea narrow and gaining elevation before departure, storks decreased ODBA and increased the absolute descent, descent angle and minimum altitude above water (Figs. 4 and 5), allowing for a safer and less demanding oversea flight and thus giving support to the departure position hypothesis. With regards to the effects of wind conditions, head winds increased air distance, cross winds and head winds demanded higher air speeds and tail winds reduced both air distance and air speeds through the sea narrow (Fig. 6). Tail winds thus exerted a supporting effect during oversea flights, consistent with their positive effects on the probability to initiate sea crossings recorded in soaring migrants in the same study area^15,28^. Interestingly, while the traversed ground distances we recorded were on average 13% longer that the actual lengths of the sea narrows at departure (thus showing a considerable adherence to the minimal routes from the realized departure locations), air distances were on average only 7% longer, with extreme examples of birds traversing air distances as short as 12.6 km oversea, that is, shorter distances than the minimum geographical length between Europe and Africa. Overall these results support the oversea wind hypothesis, in line with the prevailing observation that local winds are major determinants of sea crossing performance in soaring landbirds (e.g.^23,28,39^), and indicating that storks adjusted their movements to the conditions of the travelling environment to successfully reduce travel costs. Interestingly, despite a high variability in realized departure locations and oversea route lengths (Table S1) the average population position at departure and arrival shores revealed a route that was close to the shortest possible width across the sea straits (Fig. 1).

In summary, our study provides a prime example at high temporal resolution of how birds adjust their behavior and physiology as they interact with the changing conditions of the travelling medium, reallocating resources and modifying their movement patterns to overcome an ecological barrier.

## Methods

### Bird tagging and tag settings

During 2013 and 2014, a total of 91 white storks were captured at their nests in Southwest Spain (37°12′30.2″ N, 6°10′20.0″ W, N = 30 birds) or Southwest Germany (47° 45′ 10.8″ N, 8°56′2.4″ E; N = 61 birds; see^11^ and equipped with 54 g, solar GSM-GPS-ACC loggers (e-obs GmbH; Munich, Germany) attached as a backpack through a Teflon-nylon harness. The birds were either nestlings tagged 1–2 weeks before fledging (N = 79) or adults from the Spanish population tagged during the chick raising period (N = 12). Of the 91 tagged storks 28 individuals (24 juveniles and 4 adults) flew from Europe to Africa as part of their fall migration, allowing us to select the track portions corresponding to their southbound movements across the straits of Gibraltar (N = 28 tracks, one track per bird). The remaining birds corresponded to sedentary individuals, short-distance and intra-continental migrants and juvenile casualties whose movements were exclusively confined within the European continent, thus precluding oversea migration analysis. Total backpack weight (i.e. transmitter plus harness) was below 3% mean body mass. Transmitters recorded GPS geographical position and elevation with an accuracy of ± 3.6 m as well as speed, heading and three-axial body acceleration (for 3.8 s at 10.54 Hz) every 5 min, daily between 4:00 and 22:00 h (Central European Summer Time). Sensor information was sent via the GSM network to an internet server and stored in the online database MoveBank (https://www.movebank.org). The field procedures were approved by the Spanish CSIC Ethical Committee and the Andalusian Committee of Animal Experimentation (refs. CEBA EBD-11-24/12-39), as well as the German state authorities (Regierungspräsidium Freiburg, Reg. No. G-13/28, G-15/47, Regierungspräsidium Tübingen MPI-O-1/14, Regierung von Mittelfranken 54-2532.1-14/14, Regierung von Oberbayern 55.2-1-55-2532-22-2015, Landesuntersuchungsamt Rheinland-Pfalz G 15-20-032) to comply with national and European legislation on the protection of animals used for scientific purposes.

### Study area, migratory milestones and characterization of itineraries

The straits of Gibraltar (Fig. 1) are located between the southernmost tip of the Iberian Peninsula in Spain and the northernmost tip of Morocco, and constitute the shortest sea narrows between Europe and Africa across the Mediterranean sea (with c.a. 14 km separating the European isthmus of Tarifa from the African coast). This area is a major migratory bottleneck for soaring landbirds, which typically avoid wide bodies of water and gather in large numbers at this narrow sea passage (e.g., over 100.000 storks are annually observed between July and October on their southbound migration^19^). Individual movement tracks were decomposed into the three prime journey portions (thereafter "itineraries") that are sequentially traversed during southbound migration through the straits of Gibraltar. The three itineraries (red lines in Fig. 1) were: (i) overland itinerary in Europe (the itinerary traversed overground in the European shore, during the preparation for departure or "pre-cross"); (ii) oversea itinerary (the itinerary across the actual water narrow), and (iii) overland itinerary in Africa (the itinerary traversed overground, after arrival at the African shore or "post-cross"). The lengths of the two overland itineraries were calculated individually to match the variable length of the oversea trajectory of each bird (i.e. mean and s.e.m. 22.3 ± 1.3 km). The three itineraries combined were always traversed in a continuous, non-stop single flight (i.e. all GPS fixes showed speeds > 2 m/s) with an average length of 66.8 km. To further analyze sequential changes in trip parameters as the storks moved across the water channel, each oversea itinerary was decomposed into initial, middle and final positions overwater using the first, intermediate and last GPS fix recorded therein. In order to assess the causes and consequences of route choices during the preparation for the sea crossing, we defined the European hinterland as the land located between the parallel 36°-11′ 00″ and the Spanish shore (north and south limits respectively; see dotted area in Fig. 1). This area contained c.a. 580 Km^2^ of land, included all the shore-departure locations historically described for migrating white storks^12^ and was always traversed by the sampled birds before starting their oversea journeys. Since storks movements sequentially intersected the north boundary of the European hinterland, the European shoreline and the African shoreline, we calculated the average population position across these boundaries (yellow dots in Fig. 1). Longitudinal deviations from each track to the population mean allowed us to quantify the relative location of individuals (thereafter "location" in Km, with positive and negative numbers indicating east and west deviations respectively) at three milestones characterizing their local migratory movements, namely (i) entry location (the location of birds as they entered the European hinterland), (ii) departure location (the location of departure from the European shore) and (iii) arrival location (the location of arrival at the African shore).

### Calculation of trip parameters

For each itinerary and/or position described above, we calculated the following parameters:

- Traversed ground distance (Km): sum of leg distances between the sequential GPS fixes comprising a given itinerary.
- Minimum distance (Km): straight line (i.e. ideal minimum) distance between the initial and the final GPS fixes of a given itinerary.
- Straightness (m m^−1^): distance effectively progressed per distance unit actually traversed (minimum distance divided by traversed distance) within a given itinerary.
- Duration (min): time passed (active flight time) between the initial and the final GPS fixes of a given itinerary.
- Wind speed (Km h^−1^): speed of the wind along the bird's flight path, with positive values occurring in the direction of the bird's motion.
- Air speed (Km h^−1^): speed of the bird relative to the air in which it is flying.
- Ground speed (Km h^−1^): horizontal speed of the bird relative to the ground.
- Altitude above terrain (m): vertical distance between the bird and the surface (land or water) underneath extracted from a digital elevation model (ASTER, 30 m resolution).
- Altitude above sea level (m): vertical distance above sea level.
- Climb (m): change in flight altitude (final minus initial altitude) within a given itinerary; with positive values indicating ascent. The inverse of this variable (i.e. climb × (− 1)) is presented as descent (m).
- Climb angle (m m^−1^): rate of altitude change with respect to horizontal distance (climb divided by traversed distance), with positive values implying ascent. The inverse of this variable (i.e. climb rate × (− 1)) is presented as descent angle (m m^−1^).
- ODBA (m s^−2^): overall dynamic body acceleration, calculated as the sum of dynamic acceleration values from the three orthogonal axes of the onboard accelerometer.
- Flapping mode frequency (%): relative frequency of ACC fixes indicating a flapping flight mode within a given itinerary (classified according to^41^; see below).
- Soaring mode frequency (%): relative frequency of ACC fixes indicating a soaring flight mode within a given itinerary (classified according to^41^; see below).
- Gliding mode frequency (%): relative frequency of ACC fixes indicating a gliding flight mode within a given itinerary (classified according to^41^; see below).

In addition, for oversea itineraries we calculated air distance defined as the distance traversed in relation to the air in which the bird is flying (i.e. air speed × time). All the parameter calculations were done using ArcGis 10.1. Wind data were extracted with spatial and temporal resolutions of 2.5 × 2.5° and 6 h from the NCEP/NCAR R-1 reanalysis dataset following Kemp et al.^42^. Orthogonal zonal (u) and meridional (v) wind velocity components were obtained using the package RNCEP in R 3.2.2, calculated for each GPS fix interpolating to the pressure level nearest to storks’ flight altitude and used to calculate wind support, cross wind and airspeed following Safi et al.^43^. Raw acceleration data were converted from mV into g units using tag-specific calibration values following Flack et al.^11^. The three signals were smoothed using running means over 3.8 s, the smoothed data subtracted from the corresponding unsmoothed data, and the sum of all three axes provided a value of Overall Dynamic Body Acceleration ODBA (reflecting an individual´s overall body movements^31–33^). Flight modes were characterized every 5–10 min following Collins et al.^41^, with ACC data allowing discrimination of flapping from soaring or gliding, and the net climb recorded between sequential GPS fixes allowing discrimination between the latter two behaviors (i.e. ascent vs. descent climbs respectively defined as soaring and gliding). Part of the data used for this study are contained in the MPIO white stork lifetime tracking data^11^ and are available upon request.

### Statistical analyses

Changes in trip parameters and flight performance among itineraries were tested through Generalized Linear Mixed Models (GLMMs^44^) that considered itinerary as a fixed factor with three levels (i.e. overland in Europe pre-cross, oversea, and overland in Africa post-cross) and individual identity as a random term (thus controlling for the potential pseudoreplication associated with repeated measures from the same birds). Post-hoc contrasts allowed pairwise comparisons between itineraries when the main effect was statistically significant (i.e. P ≤ 0.05). Given the expected lack of soaring oversea, in order to make flight parameters more comparable among itineraries we constructed a second set of GLMMs where the speed (air and ground), climb (absolute and climb angle) and ODBA were tested using only the track portions corresponding to active flapping. Additional GLMMs were built to test changes in flight parameters within the oversea itinerary, but here models considered individual position overwater (i.e. initial, middle and final positions) as a fixed factor. The causes and consequences of route choices during the preparation for the sea crossing were tested through Linear Regression Models LMs that assessed the simultaneous effects of prior locations and zonal wind components on subsequent milestone locations along the migratory route. Finally, we used Generalized Linear Models GLMs to analyze the potential effects that the initial position at the start of the oversea journey (i.e. length of the sea narrow and altitude at departure) plus the wind conditions experienced on route (i.e. wind support and squared cross wind overwater) could exert on critical components of the oversea migration including traversed ground and air distances, time, ground and air speeds, ODBA, minimum altitude overwater, climb and climb angle. All the models were built using SAS 9.3. (SAS Institute, Cary NC). Additional details on models structure and data transformation are provided in the captions of Tables S2–S6.

## Supplementary information

Supplementary Information.

## Data Availability

All data generated during this study are included in this published article, its Supplementary Information files, or are available from the corresponding author on reasonable request.
