# Supplementary material for: Overland and oversea migration of white storks through the water barriers of the straits of Gibraltar
Source: Sci Rep. 2020 Dec 1;10:20760. doi: 10.1038/s41598-020-77273-x (PMC7708975; doi:10.1038/s41598-020-77273-x)
Supplement: Supplementary file 1 — Supplementary Information. [file 41598_2020_77273_MOESM1_ESM.pdf]

SUPPLEMENTARY INFORMATION

Overland and oversea migration of white storks through the water barriers of the straits of Gibraltar

Julio Blas, Reyes Salas, Andrea Flack, Fernando Torres-Medina, Fabrizio Sergio, Martin Wikelski and

Wolfgang Fiedler

Figure S1. Sequential changes in flight modes overland and overwater during white stork migration across the strait of Gibraltar. Panel A shows relative frequencies of flapping, soaring and gliding flight modes within and among itineraries overland in Europe pre-cross (EUR), overwater across the sea strait (SEA) and overland in Africa post-cross (AFR). Panel B shows chronological changes in flight modes (ACC+GPS fixes recorded every 5 minutes) along the complete route of one illustrative individual in relation flight altitude (Y-axis) and distance to departure shore location (X-axis).

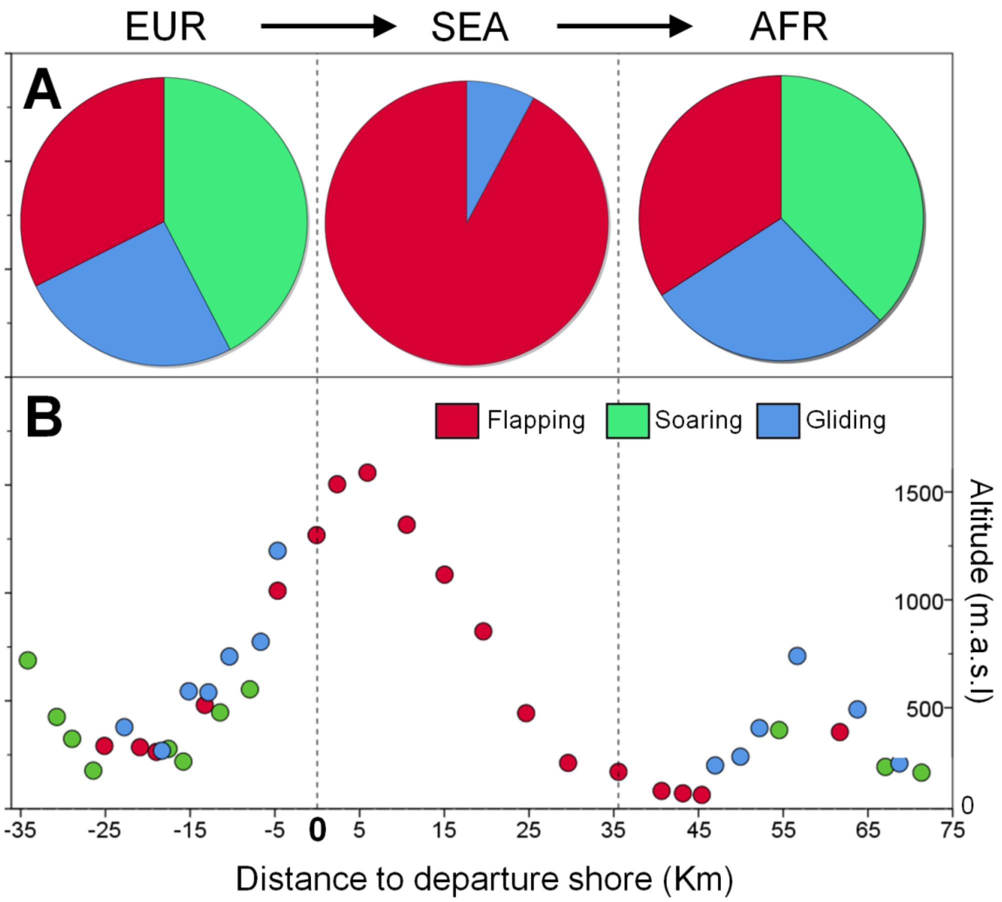

Figure S2. Sequential changes in trip parameters (panels a-f) along the oversea itineraries (X-axis) traversed by white storks during southbound migration across the strait of Gibraltar. Each itinerary was decomposed into initial, middle and final positions (INIsea, MIDsea and FINsea respectively) using the first, intermediate and last GPS fix of the overwater tracks. Asteriscs * indicate statistically significant differences (P<0.05) between sequential positions. Filled circles and error bars represent means and standard errors.

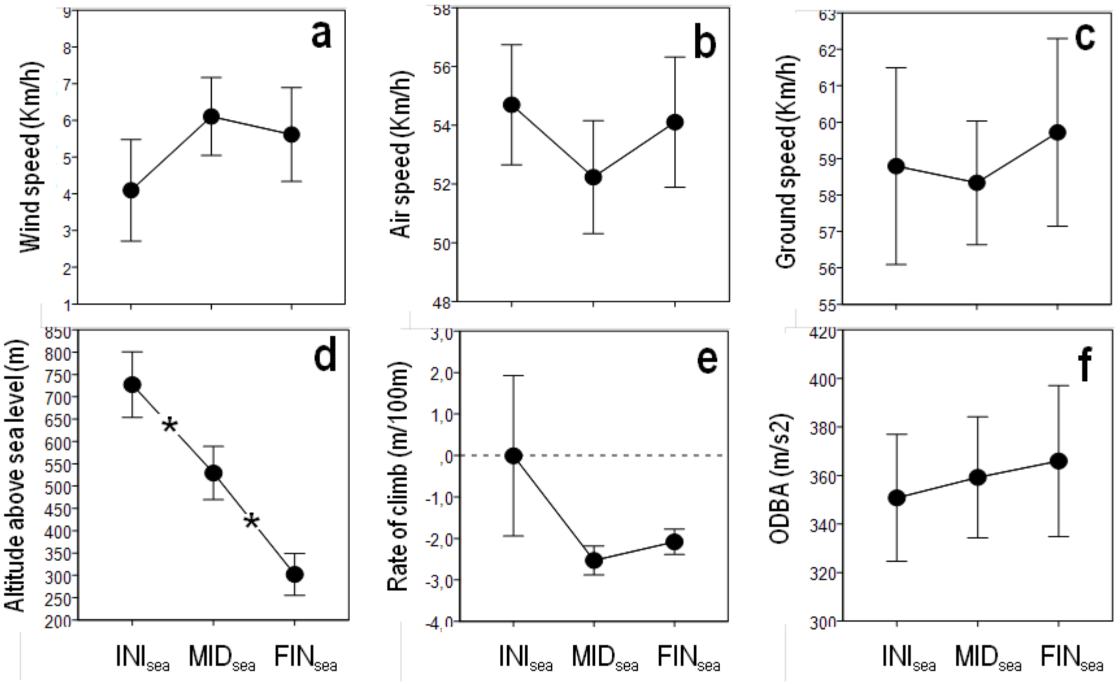

Table S1. Description and summary statistics of the trip and flight parameters characterizing overland and overwater migration of white storks through the strait of Gibraltar.

Parameter	Description		Itinerary†		
			EUR	SEA	AFR
**Ground distance (Km)**	Summatory of leg distances between the sequential gps fixes comprising a given itinerary.	(a)	**22.6** ± 1.2 (12.5; 38.9)	**22.3** ± 1.3 (15.1; 38.2)	**21.9** ± 1.2 (15.4; 38.2)
**Minimum distance (Km)**	Straight line distance between the initial and the final gps fixes of a given itinerary.	(a)	**20.4** ± 1.1 (10.6; 34.2)	**21.8** ± 1.2 (14.7; 37.4)	**20.6** ± 1.1 (14.3; 34.7)
**Air distance (Km)**	Distance traversed in relation to the air in which the bird is flying (air speed x time).	(a)	-	**21.0** ± 1.2 (12.6; 37.1)	-
**Straightness (m/100m)**	Distance effectively progressed per distance unit actually traversed (minimum distance divided by traversed distance) within a given itinerary.	(a)	**90.5** ± 1.6 (63.4; 98.9)	**98.2** ± 0.3 (93.3; 100)	**94.2** ± 0.5 (89.5; 99.8)
**Duration (min)**	Time passed (active flight time) between the initial and the final GPS fixes of a given itinerary.	(a)	**48** ± 3.4 (15; 101)	**23.5** ± 1.5 (14; 44)	**41.7** ± 2.2 (19; 63)
**Wind speed (Km/h)**	Speed of the wind along the bird's flight path (positive values occur in the direction of the bird's motion).	(a)	**1.4** ± 0.8 (-4.5; 14.5)	**5.4** ± 0.9 (-5.9; 15.9)	**1.6** ± 1.1 (-9.6; 11.5)
		(b)	**10.8** ± 0.9 (2.9; 24.2)	**11.0** ± 0.9 (4.5; 26.6)	**11.7** ± 1.1 (5.2; 26.9)
**Air speed (Km/h)**	Speed of the bird relative to the air in which it is flying.	(a)	**44.3** ± 1.1 (34.9; 59.1)	**54.1** ± 1.3 (43.2; 65.9)	**44.7** ± 1.1 (32.8; 60.4)
		(b)	**48.6** ± 1.7 (29.9; 62.5)	**54.7** ± 1.5 (37.3; 66.2)	**50.2** ± 2.3 (33.5; 73.5)
**Ground speed (Km/h)**	Horizontal speed of the bird relative to the ground.	(a)	**45.7** ± 1.4 (30.6; 60.4)	**59.6** ± 1.2 (46; 70.3)	**46.3** ± 1.1 (36.7; 57.4)
		(b)	**52.7** ± 1.7 (30.2; 62.1)	**59.9** ± 1.5 (44.9; 71.5)	**50.5** ± 2.9 (22.7; 84.1)
**Altitude above terrain (m)**	Vertical distance between the bird and the (land or water) surface underneath.	(a)	**610.2** ± 57.2 (124; 1453.4)	**511.5** ± 56.4 (60.6; 1073.3)	**408.3** ± 28.5 (154.6; 772.5)
**Altitude above sea level (m)**	Vertical distance above sea level.	(a)	**692.9** ± 61.5 (128.5; 1536.5)	**511.5** ± 56.4 (60.6; 1073.3)	**566.3** ± 30.1 (268.8; 901.1)
**Climb (m)**	Change in flight altitude (final minus initial altitude) within a given itinerary, with negative values implying a descent.	(a)	**289.8** ± 90.3 (-374.7; 1290.5)	**-424.9** ± 56.5 (-1332.3; -17.5)	**402.4** ± 74.5 (-183.2; 1522.2)
**Climb angle (m/100m)**	Altitude gained per distance travelled over the ground (negative values imply a descent).	(a)	**1.5** ± 0.5 (-2.3; 9.0)	**-1.8** ± 0.1 (-3.5; -0.1)	**1.8** ± 0.4 (-1.2; 5.8)
		(b)	**-0.7** ± 2.0 (-13.4; 42.9)	**-1.9** ± 0.2 (-4.3; 0.4)	**-0.1** ± 1.0 (-8.9; 14.1)
**ODBA (m/s2)**	Overall dynamic body acceleration; sum of acceleration values from the three orthogonal axes of the onboard accelerometer.	(a)	**164.8** ± 16.4 (57.6; 497.3)	**344** ± 17.9 (91.7; 477.8)	**160.6** ± 13.2 (46.2; 293.7)
		(b)	**327.5** ± 15.7 (172.8; 497.3)	**376.4** ± 16.6 (163.9; 495.8)	**326.4** ± 14.6 (207.7; 476.0)
**Flap frequency (%)**	Relative frequency of ACC fixes indicating flapping flight within a given itinerary.	(a)	**33.8** ± 4.1 (0; 100)	**92.1** ± 3.4 (50; 100)	**32.4** ± 4.5 (0; 77.8)
**Soar frequency (%)**	Relative frequency of ACC fixes indicating soaring flight within a given itinerary.	(a)	**37.5** ± 3.45 (0; 71.4)	**0.0** ± 0 0	**42.4** ± 5.4 (0; 100)
**Glide frequency (%)**	Relative frequency of ACC fixes indicating gliding flight within a given itinerary.	(a)	**27.9** ± 2.9 (0; 71.4)	**7.9** ± 3.4 (0; 50)	**25.3** ± 5.1 (0; 100)

†European itineraries (EUR) were the overground tracks traversed during preparation for departure in the European shore; sea itineraries (SEA) were the oversea tracks traversed across the water barrier; African itineraries (AFR) were the overground tracks traversed upon arrival to the African shore. The reported values and mean, standard errors and range (minimum and maximum, within brackets). (a) Summary statistics calculated using all the gps fixes within a given itinerary, regardless of the flight mode; (b) summary statistics calculated using only GPS fixes corresponding to flapping flight.

Table S2. Results from the Generalized Linear Mixed Models assessing changes in trip parameters across the three main itineraries (overland and overwater) comprising southbound migration across the strait of Gibraltar. Individual identity was fitted as a random term to all models. EUR = overland itinerary in Europe, before reaching the departure shore; SEA = overwater itinerary across the sea strait; AFR= overland itinerary in Africa, after reaching the arrival shore.

Variable •	Parameter estimate ± s.e.m.	Test	*P*	Post-hoc contrasts		
				Groups	Test	*P*
**A. Dependent: Traversed distance (m)††**						
Itinerary (EUR)	0.04 ± 0.03	$F_{2,54}$=0.72	0.49	–	–	–
Itinerary (SEA)	0.01 ± 0.03					
Intercept	9.96 ± 0.05					
**B. Dependent: Minimun distance (m)††**						
Itinerary (EUR)	-0.004 ± 0.037	$F_{2,54}$=1.53	0.23	–	–	–
Itinerary (SEA)	0.053 ± 0.037					
Intercept	9.901 ± 0.054					
**C. Dependent: Straightness (m·m^{-1})§**						
Itinerary (EUR)	-0.50 ± 0.16	$F_{2,54}$=25.27	***	EUR vs SEA	$F_{1,54}$=48.95	***
Itinerary (SEA)	1.17 ± 0.25			EUR vs AFR	$F_{1,54}$=9.59	**
Intercept	2.79 ± 0.14			SEA vs AFR	$F_{1,54}$=21.92	***
**D. Dependent: Duration (min)†**						
Itinerary (EUR)	6.3 ± 2.3	$F_{2,54}$=61.33	***	EUR vs SEA	$F_{1,54}$=113.79	***
Itinerary (SEA)	-18.1 ± 2.3			EUR vs AFR	$F_{1,54}$=7.60	**
Intercept	41.7 ± 2.5			SEA vs AFR	$F_{1,54}$=62.58	***
**E. Dependent: Wind speed (Km·h^{-1})†**						
Itinerary (EUR)	-0.21 ± 1.09	$F_{2,54}$=8.64	***	EUR vs SEA	$F_{1,54}$=13.66	***
Itinerary (SEA)	3.81 ± 1.09			EUR vs AFR	$F_{1,54}$=0.04	0.84
Intercept	1.63 ± 0.93			SEA vs AFR	$F_{1,54}$=12.22	***
**F. Dependent: Air speed (Km·h^{-1})†**						
Itinerary (EUR)	-0.36 ± 1.43	$F_{2,54}$=30.56	***	EUR vs SEA	$F_{1,54}$=47.55	***
Itinerary (SEA)	9.47 ± 1.43			EUR vs AFR	$F_{1,54}$=0.07	0.99
Intercept	44.66 ± 1.14			SEA vs AFR	$F_{1,54}$=44.08	***
**G. Dependent: Ground speed (Km·h^{-1})†**						
Itinerary (EUR)	-0.68 ± 1.65	$F_{2,54}$=44.81	***	EUR vs SEA	$F_{1,54}$=70.50	***
Itinerary (SEA)	13.18 ± 1.65			EUR vs AFR	$F_{1,54}$=0.17	0.68
Intercept	46.36 ± 1.27			SEA vs AFR	$F_{1,54}$=63.76	***
**H. Dependent: Altitude above terrain (m)†**						
Itinerary (EUR)	201.93 ± 52.86	$F_{2,54}$=7.30	**	EUR vs SEA	$F_{1,54}$=3.48	0.06
Itinerary (SEA)	103.36 ± 52.86			EUR vs AFR	$F_{1,54}$=14.59	***
Intercept	408.21 ± 49.22			SEA vs AFR	$F_{1,54}$=3.82	0.05
**I. Dependent: Altitude above sea level (m)†**						
Itinerary (EUR)	126.57 ± 54.86	$F_{2,54}$=5.75	**	EUR vs SEA	$F_{1,54}$=10.92	**
Itinerary (SEA)	54.75 ± 54.86			EUR vs AFR	$F_{1,54}$=5.32	*
Intercept	566.32 ± 51.26			SEA vs AFR	$F_{1,54}$=1.00	0.32
**J. Dependent: Climb (m)†**						
Itinerary (EUR)	-112.54 ± 106.16	$F_{2,54}$=35.72	***	EUR vs SEA	$F_{1,54}$=45.33	***
Itinerary (SEA)	-827.25 ± 106.16			EUR vs AFR	$F_{1,54}$=1.12	0.29
Intercept	402.39 ± 75.07			SEA vs AFR	$F_{1,54}$=60.72	***

**K. Dependent: Climb angle (m·m⁻¹)†, #**						
Itinerary (EUR)	-0.05 ± 0.05	$F_{2,54}$=45.45	***	EUR vs SEA	$F_{1,54}$=59.42	***
Itinerary (SEA)	-0.44 ± 0.051			EUR vs AFR	$F_{1,54}$=1.01	0.32
Intercept	-1.26 ± 0.04			SEA vs AFR	$F_{1,54}$=75.91	***
**L. Dependent: ODBA (m·s⁻²)†**						
Itinerary (EUR)	4.14 ± 18.31	$F_{2,54}$=65.39	***	EUR vs SEA	$F_{1,54}$=95.82	***
Itinerary (SEA)	183.36 ± 18.31			EUR vs AFR	$F_{1,54}$=0.05	0.82
Intercept	160.65 ± 15.95			SEA vs AFR	$F_{1,54}$=100.30	***
**M. Dependent: Flapping frequency (%)¶**						
Itinerary (EUR)	-0.18 ± 0.20	$F_{2,54}$=32.31	***	EUR vs SEA	$F_{1,54}$=64.03	***
Itinerary (SEA)	3.66 ± 0.48			EUR vs AFR	$F_{1,54}$=0.79	0.38
Intercept	-0.62 ± 0.17			SEA vs AFR	$F_{1,54}$=57.73	***
**N. Dependent: Gliding frequency (%)¶**						
Itinerary (EUR)	0.39 ± 0.22	$F_{2,54}$=10.72	***	EUR vs SEA	$F_{1,54}$=20.66	***
Itinerary (SEA)	-1.79 ± 0.49			EUR vs AFR	$F_{1,54}$=3.05	0.09
Intercept	-1.25 ± 0.18			SEA vs AFR	$F_{1,54}$=13.50	***
**O. Dependent: Soaring frequency (%)††**						
Itinerary (EUR)	-0.10 ± 0.14	$F_{2,54}$=134.06	***	EUR vs SEA	$F_{1,54}$=235.71	***
Itinerary (SEA)	-3.71 ± 0.14			EUR vs AFR	$F_{1,54}$=0.55	0.46
Intercept	3.71 ± 0.11			SEA vs AFR	$F_{1,54}$=250.15	***

•Values in parentheses indicate the sequential levels of a categorical variable (the parameter estimates for the overland itinerary in Africa are included in the intercept). ††Generalized linear mixed model with Negative Binomial errors and a logarithmic link function. †General linear mixed model with Gaussian errors and identity link function. # Log(x+0.04) transformed response. § Generalized linear mixed model with Beta errors and logistic link function. ¶ Generalized linear mixed model with Binomial errors and logistic link function. Asterisks indicate the level of statistical significance (*P<0.05; **P<0.01; *** P<0.001)

Table S3. Results from the Generalized Linear Mixed Models GLMMs testing changes in movement parameters during flapping flight overland and oversea. Individual identity was fitted as a random term to all models. EUR = overland in Europe (before reaching the departure shore); SEA = overwater; AFR= overland in Africa (after reaching the arrival shore).

Variable •	Parameter estimate ± s.e.m.	Test	*P*	Post-hoc contrasts		
				Groups	Test	*P*
**A. Dependent: Wind speed (Km·h⁻¹)†**						
Itinerary (EUR)	-0.6676 ± 0.3663					
Itinerary (SEA)	-0.3531 ± 0.3657	$F_{2,45}$=1.66	0.20	–	–	–
Intercept	11.3531 ± 0.9083					
**B. Dependent: Climb angle (m·m⁻¹)†**						
Itinerary (EUR)	0.0009 ± 0.0134					
Itinerary (SEA)	-0.0086 ± 0.017	$F_{2,45}$=0.67	0.52	–	–	–
Intercept	-0.0196 ± 0.0170					
**C. Dependent: Air speed (Km·h⁻¹)† #**						
Intercept	1.695 ± 0.017			EUR vs SEA	$F_{1,45}$=8.22	**
Itinerary (EUR)	-0.015 ± 0.019	$F_{2,45}$=4.36	*	EUR vs AFR	$F_{1,45}$=0.62	0.43
Itinerary (SEA)	0.038 ± 0.019			SEA vs AFR	$F_{1,45}$=3.80	*
**D. Dependent: Ground speed (Km·h⁻¹)†**						
Itinerary (EUR)	50.96 ± 2.14			EUR vs SEA	$F_{1,45}$=8.22	**
Itinerary (SEA)	1.74 ± 2.59	$F_{2,45}$=7.9	***	EUR vs AFR	$F_{1,45}$=0.45	0.5
Intercept	8.83 ± 2.55			SEA vs AFR	$F_{1,45}$=12.02	**
**E. Dependent: ODBA (m·s⁻²)†**						
Itinerary (EUR)	326.21 ± 16.93			EUR vs SEA	$F_{1,45}$=5.66	*
Itinerary (SEA)	0.65 ± 22.12	$F_{2,45}$=3.79	*	EUR vs AFR	$F_{1,45}$=0.001	0.97
Intercept	50.18 ± 21.62			SEA vs AFR	$F_{1,45}$=5.39	*

•Values in parentheses indicate the sequential levels of a categorical variable (the parameter estimates for the overland itinerary in Africa are included in the intercept). †General linear mixed model with Gaussian errors and identity link function. # Log transformed response. Asterisks indicate the level of statistical significance (*P≤0.05; **P≤0.01; *** P≤0.001)

Table S4. Results from the Generalized Linear Mixed Models assessing changes in trip parameters within the oversea itineraries. The overwater tracks of white storks were decomposed into initial, middle and final positions (INIsea, MIDsea and FINsea respectively) using the first, intermediate and last GPS fix of each itinerary. Individual identity was fitted as a random term to all models.

Variable•	Parameter estimate ± s.e.m.	Test	*P*	Post-hoc contrasts		
				Groups	Test	*P*
**A. Dependent: Wind speed ($Km·h^{-1}$)††, §**						
Oversea position (INI)	-0.14 ± 0.09	$F_{2,54}$=0.66	0.20	–	–	–
Oversea position (MID)	-0.00 ± 0.09					
Intercept	6.49 ± 0.10					
**B. Dependent: Air speed ($Km·h^{-1}$)††**						
Oversea position (INI)	0.011 ± 0.05	$F_{2,54}$=0.37	0.69	–	–	–
Oversea position (MID)	-0.03 ± 0.05					
Intercept	3.99 ± 0.03					
**C. Dependent: Ground speed ($Km·h^{-1}$)††**						
Oversea position (INI)	-0.017 ± 0.06	$F_{2,54}$=0.66	0.89	–	–	–
Oversea position (MID)	-0.02 ± 0.06					
Intercept	4.09 ± 0.04					
**D. Dependent: Altitude above sea level (m)††**						
Oversea position (INI)	0.99 ± 0.09	$F_{2,54}$=57.23	***	INI *vs* MID	$F_{1,54}$= 16.26	***
Oversea position (MID)	0.62 ± 0.09			INI *vs* END	$F_{1,54}$=112.47	***
Intercept	5.44 ± 0.15			MID *vs* END	$F_{1,54}$= 43.31	***
**E. Dependent: Descent angle ($m·m^{-1}$)¥, #**						
Oversea position (INI)	-0.08 ± 0.06	$F_{2,54}$=1.44	0.25	–	–	–
Oversea position (MID)	0.02 ± 0.06					
Intercept	0.08 ± 0.046					
**F. Dependent: ODBA ($m·s^{-2}$)†, ¶**						
Oversea position (INI)	-19793 ± 19832	$F_{2,44}$=0.74	0.48	–	–	–
Oversea position (MID)	-21911 ± 20375					
Intercept	159235 ± 16587					

•Values in parentheses indicate the sequential levels of a categorical variable (the parameter estimate for the final position oversea is included in the intercept). ††Generalized linear mixed model with Negative Binomial errors and a logarithmic link function. # Transformed (x+0.5) response. § Transformed $(x+20)^2$ response. †General linear mixed model with Gaussian errors and identity link function. ¥Generalized linear mixed model with Beta errors and a logistic link function. ¶N=74 observations from 28 individuals. Asterisks indicate the level of statistical significance (*P<0.05; **P<0.01; *** P<0.001)

Table S5.

Results from the LMs predicting the location of migrating storks in the European and African shores in relation to their previous locations and concurrent zonal winds. Location represents east or west deviations (positive and negative values respectively) from the mean population longitude at the three milestones characterizing oversea migration routes: the European hinterland, the European (departure) shore, and the African (arrival) shore.

Variable	Parameter estimate ± s.e.m.	Test	*P*	ΔAIC§	R^2
**A. Dependent: European shore (departure location, m)**				-10.4	0.42
European hinterland (entry location, m)	0.42 ± 0.13	$F_{1,25}$=11.31	**	7.8	
European hinterland (entry UV-wind, m/s)	1220.33 ± 486.47	$F_{1,25}$=6.29	*	3.7	
Intercept	-743.14 ± 1230.57				
**B. Dependent: African shore (arrival location, m)**				-11.4	0.44
European hinterland (entry location, m)	0.47 ± 0.17	$F_{1,25}$=7.38	*	4.6	
European hinterland (entry UV-wind, m/s)	2276.47 ± 662.15	$F_{1,25}$=11.82	**	8.2	
Intercept	-1386.33 ± 1674.94				
**C. Dependent: African shore (arrival location, m)**				-40.6	0.80
European shore (departure location, m)	1.09 ± 0.14	$F_{1,25}$=63.08	***	32.6	
European shore (departure UV-wind, m/s)	909.66 ± 435.21	$F_{1,25}$=4.37	*	1.9	
Intercept	-712.20 ± 1023.85				

§ For the whole model (first line of each model) we report the change in AIC in relation to the null model (containing only the intercept). For each individual variable, we report the change in AIC that would derive from the removal of the variable from the maximal model (i.e. negative values imply that the removal of the variable improved the fit of the model). Asterisks indicate the level of statistical significance (*P<0.05; **P<0.01; *** P<0.001)

Table S6. Results from the Generalized Linear Models GLM analyzing migration performance overwater. GLM tested the effects of departure position at the start of the oversea itinerary (i.e. length of the water narrow and altitude at shore departure) and the wind conditions experienced on route (i.e. average wind support and cross winds) on several components of storks migration overwater.

	Response	Independent	Parameter estimate ± s.e.m.	Test	*P*	ΔAIC§
**A.**	**Ground distance oversea (m)†**	Intercept	1758.0 ± 1240.9	–	–	-72.0
		Length of the water narrow	1.055 ± 0.056	$F_{1,23}$=344.88	***	74.4
		Altitude at departure	0.377 ± 1.013	$F_{1,23}$=0.14	0.712	-3.0
		Wind support on route	-11.356 ± 76.086	$F_{1,23}$=0.02	0.882	-3.2
		Cross wind^2 on route	-7.142 ± 4.103	$F_{1,23}$=3.03	0.095	0.3
**B.**	**Air distance oversea (m)†**	Intercept	5457.943 ± 1802.462	–	–	-49.0
		Length of the water narrow	0.88 ± 0.0825	$F_{1,23}$=113.75	***	45.0
		Altitude at departure	1.1045 ± 1.4725	$F_{1,23}$=0.56	0.461	-2.5
		Wind support on route	-398.352 ± 110.5166	$F_{1,23}$=12.99	**	7.6
		Cross wind^2 on route	-2.8273 ± 5.9604	$F_{1,23}$=0.23	0.64	-4.6
**C.**	**Time oversea (min)†**	Intercept	4.9914 ± 3.38	–	–	-25.1
		Length of the water narrow	0.0011 ± 0.0002	$F_{1,23}$=48.26	***	28.5
		Altitude at departure	-0.0016 ± 0.0028	$F_{1,23}$=0.35	0.559	-2.8
		Wind support on route	-0.0368 ± 0.2072	$F_{1,23}$=0.03	0.860	-3.1
		Cross wind^2 on route	-0.0187 ± 0.0112	$F_{1,23}$=2.79	0.108	0.0
**D.**	**Ground speed oversea (Km·h^{-1})†**	Intercept	54.1286 ± 4.5252	–	–	1.9
		Length of the water narrow	-0.0001 ± 0.0002	$F_{1,23}$=0.17	0.683	-3.0
		Altitude at departure	0.0058 ± 0.0037	$F_{1,23}$=2.5	0.127	-0.3
		Wind support on route	0.1797 ± 0.2775	$F_{1,23}$=0.42	0.523	-2.7
		Cross wind^2 on route	0.0274 ± 0.015	$F_{1,23}$=3.35	0.080	0.6
**E.**	**Air speed oversea (Km·h-1) †**	Intercept	54.5983 ± 4.4806	–	–	0.6
		Length of the water narrow	-0.0001 ± 0.0002	$F_{1,23}$=0.12	0.729	-3.0
		Altitude at departure	0.0051 ± 0.0037	$F_{1,23}$=1.92	0.18	-0.9
		Wind support on route	-0.8021 ± 0.2747	$F_{1,23}$=8.52	**	5.6
		Cross wind^2 on route	0.034 ± 0.0148	$F_{1,23}$=5.26	*	2.6
**F.**	**Mean ODBA oversea (m·s^{-2})†**	Intercept	315.6263 ± 61.2003	–	–	-1.0
		Length of the water narrow	0.0055 ± 0.0028	$F_{1,23}$=3.92	0.059	1.2
		Altitude at departure	-0.134 ± 0.05	$F_{1,23}$=7.19	*	4.4
		Wind support on route	1.0222 ± 3.7524	$F_{1,23}$=0.07	0.787	-3.1
		Cross wind^2 on route	0.1643 ± 0.2024	$F_{1,23}$=0.66	0.425	-2.4
**G.**	**Minimun altitude oversea (m)†**	Intercept	425.5773 ± 91.1055	–	–	-33.3
		Length of the water narrow	-0.0265 ± 0.0042	$F_{1,23}$=40.49	***	25.3
		Altitude at departure	0.5363 ± 0.0744	$F_{1,23}$=51.92	***	29.9
		Wind support on route	-1.2079 ± 5.5861	$F_{1,23}$=0.05	0.830	-3.1
		Cross wind^2 on route	0.2253 ± 0.3013	$F_{1,23}$=0.56	0.462	-2.5
**H.**	**Descent oversea (m)†**	Intercept	-422.348 ± 89.3277	–	–	-44.2
		Length of the water narrow	0.0262 ± 0.0041	$F_{1,23}$=41.17	***	25.6
		Altitude at departure	0.4639 ± 0.073	$F_{1,23}$=40.41	***	25.2
		Wind support on route	1.1866 ± 5.4771	$F_{1,23}$=0.05	0.828	-3.1
		Cross wind^2 on route	-0.2131 ± 0.2954	$F_{1,23}$=0.52	0.470	-2.6
**I.**	**Descent angle oversea (m·m^{-1})††**	Intercept	-5.5634 ± 0.3397	–	–	-20.9

Length of the water narrow	0.0000 ± 0.0000	$F_{1,23}$=0.98	0.332	-2.0
Altitude at departure	0.0014 ± 0.0003	$F_{1,23}$=25.04	***	18.2
Wind support on route	0.0211 ± 0.0189	$F_{1,23}$=1.25	0.274	-1.7
Cross wind^2 on route	-0.0002 ± 0.001	$F_{1,23}$=0.06	0.812	-3.1

†General linear model with Gaussian errors and identity link function. ††Generalized linear model with Gamma errors and a logarithmic link function. § For the whole model (first line of each model) we report the change in AIC in relation to the null model (containing only the intercept). For each individual variable, we report the change in AIC that would derive from the removal of the variable from the maximal model (i.e. negative values imply that the removal of the variable improved the fit of the model). Asterisks indicate the level of statistical significance (*P<0.05; **P<0.01; *** P<0.001)
